# The complete chloroplast genome of *Cymbidium floribundum* var. *pumilum* (Orchidaceae)

**DOI:** 10.1080/23802359.2019.1678419

**Published:** 2019-10-18

**Authors:** Ye Ai, Tai-Xiang Xie, Juan Chen, Jie Zhou, Ming-Kun Chen, Zhong-Jian Liu

**Affiliations:** Key Laboratory of National Forestry and Grassland Administration for Orchid Conservation and Utilization at College of Landscape Architecture, Fujian Agriculture and Forestry University, Fuzhou, China

**Keywords:** *Cymbidium floribundum* var. *pumilum*, orchid, chloroplast genome, phylogenetic analysis

## Abstract

*Cymbidium floribundum* var. *pumilum* is an epiphytic orchid distributed in the southern China. It has a high ornamental value and always be used as a hybrid parent. In this study, we obtained a complete chloroplast genome of *C. floribundum* var. *pumilum* from BGISEQ-500 sequencing data. The total chloroplast genome was 155,291 bp in length, consisting of a large single copy region (LSC 84,415 bp), a small single copy region (SSC 17,484 bp), and two inverted repeat regions (IRA and IRB 26,696 bp). The complete chloroplast genome contains 139 genes, including 80 protein-coding genes, 38 transfer RNA (tRNA) genes, and 8 ribosomal RNA (rRNA) genes. In addition, the phylogenetic analysis indicates that *C. floribundum* var. *pumilum* was sister to section *Geocymbidium*, section *Pachyrhizanthe* and section *Jensoa.* The chloroplast genome will contribute to establish an effective conservation strategy for *C. floribundum* var. *pumilum*.

The genus *Cymbidium* (Orchidaceae) are terrestrial, epiphytic or rarely lithophytic, consisting of three subgenera, divided into ten sections and comprising approximately 55 species are found in the Asia with over 49 species distributed in China (Liu et al. 2006, 2009). The species of *C. floribundum* was first reported by Lindl in1833, and it is an epiphytic orchid usually grows on trees or rocks at altitudes of 100–3300 m in the subtropics and tropical areas of Asia (Liu et al. 2006; Chen 2011). However, the species of *C. pumilum* was originally published as an independent similar species not a variant of *C. floribundum* by Rolfe in 1907. Until 1980, it was merged into a variant of *C. floribundum* by Wu et al. Then, the *C. floribundum* var. *pumilum* was directly classified into the *C. floribundum* by Du Puy et al. in 1988 (Liu et al. 2006). Nonetheless, we compared the morphology and firmly believe that the *C. floribundum* var. *pumilum* is a variant of the *C. floribundum*. Therefore, we reported the complete chloroplast genome of *C. floribundum* var. *pumilum*, in order to better understand the relationship between *C. floribundum* var. *pumilum* and related genera.

In this study, the samples of *C. floribundum* var. *pumilum* were collected from Gushan Mountain Scenic Area in Fuzhou, Fujian Province, China (26°03'N, 119°24'E) and the specimens were kept in the Herbarium of Fujian Agriculture and Forestry University (specimen code DHL-GS). The total genomic DNA of *C. floribundum* var. *pumilum* was extracted from fresh leaves using a modified CTAB method (Doyle and Doyle 1987), and then sequenced by the BGISEQ-500 platform.

The complete chloroplast genome of *C. floribundum* var. *pumilum* was assembled by the GetOrganelle pipe-line (Jin et al. 2018). Then, we performed the annotation work by using the Geneious R11.15 (Kearse et al. 2012) with the chloroplast genome of *C. macrorhizon* (NC_029713) and *C. lancifolium* (NC_029712) as reference sequences. Finally, we obtained a complete chloroplast genome of *C. floribundum* var. *pumilum* and submitted to GenBank with accession number MN_173778.

The total chloroplast genome of *C. floribundum* var. *pumilum* is 155,291 bp in length and has a GC content of 36.8%. It contains a large single copy (LSC) region of 84,415 bp, a small single copy (SSC) region of 17,484 bp, and two inverted repeat regions (IRA and IRB) of 26,696 bp. Besides, the complete chloroplast genome of *C. floribundum* var. *pumilum* contains 139 genes, including 80 protein-coding genes, 38 tRNA genes, and 8 rRNA genes.

In order to investigate the phylogenetic position of *C. floribundum* var. *pumilum*, a phylogenetic tree was constructed by RAxML (Stamatakis 2014) with 1000 ultrafast bootstrap (UFBoot) replicates (Minh et al. 2013; Chernomor et al. 2016) based on 12 complete chloroplast genome sequences of *Cymbidium* (*C. tortisepalum*, *C. kanran*, *C. lancifolium*, *C. floribundum* var. *pumilum*, *C. sinense*, *C. goeringii*, *C. ensifolium*, *C. faberi*, *C. macrorhizon*, *C. mannii*, *C. aloifolium*, *C. tracyanum*) and 2 other genera (*Oncidium sphacelatum*, *Eulophia zollingeri*) as outgroups. The results showed that *C. floribundum* var. *pumilum* (from section *Floribunde*) was sister to the *C. goeringii*, *C. ensifolium*, *C. tortisepalum*, *C. faberi*, *C. kanran*, *C. sinense* (from section *Jensoa*), *C. lancifolium* (from section *Geocymbidium*) and *C. macrorhizon* (from section *Pachyrhizanthe*) with 100% bootstrap support (Figure 1).

**Figure 1.**
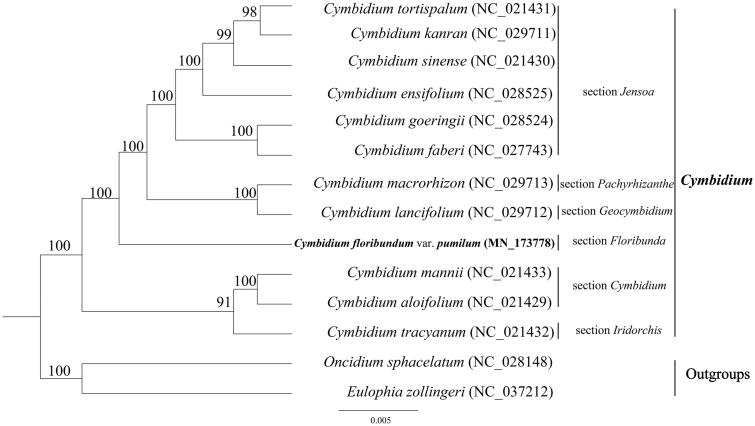
A phylogenetic tree constructed based on 12 complete chloroplast genome sequences of *cymbidium* (C. tortisepalum, C. ensifolium, C. kanran, C. aloifolium, C. faberi, C. tracyanum, C. lancifolium, C. floribundum var. pumilum, C. sinense, C. mannii, C. macrorhizon) and two other genera (*Oncidium sphacelatum, Eulophia zollingeri*) as outgroups, all of them downloaded from NCBI GenBank.
